# A study of etiology and asthma risks in infants and toddlers hospitalized for recurrent or persistent wheezing

**DOI:** 10.3389/fped.2025.1521346

**Published:** 2025-03-31

**Authors:** Qiuwei Yi, Shanshan Liu, Jingjing Qiao, Yulan Chen, Lu Huang, Yuejie Zheng, Yanmin Bao, Kunling Shen

**Affiliations:** ^1^ Respiratory Department, Beijing Children Hospital, Capital Medical University, China National Clinical Research Center of Respiratory Diseases, National Center for Children Health, Beijing, China; ^2^ Respiratory Department, Shenzhen Children’s Hospital, Shenzhen, China; ^3^ Clinical Research Laboratory, Shenzhen Children’s Hospital, Shenzhen, China

**Keywords:** infants, young children, recurrent wheezing, persistent wheezing, etiology, infantile asthma, risk factors

## Abstract

**Objective:**

To study the etiology of recurrent and persistent wheezing in infants and toddlers, and identify the risk factors for infantile asthma.

**Methods:**

We retrospectively analyzed the data of 612 children (aged 0–36 months) hospitalized for recurrent or persistent wheezing between 2019.09.01 and 2022.08.31. We comparatively analyzed their clinical, laboratory, imaging, and lung-examination data between different groups. Multivariate logistic regression analysis was used to identify risk factors for asthma.

**Results:**

The etiologies of recurrent and persistent wheezing significantly differed (*P* < 0.05). The top 3 causes of recurrent wheezing were viral infections (48.4%), infantile asthma (28.0%), and protracted bacterial bronchitis (PBB; 9.5%), whereas the top 3 causes of persistent wheezing were PBB (67.5%), tracheomalacia (22.2%), and infantile asthma (15.9%). The distribution of etiologies varied by age. Bronchoscopy was performed for 181 children, and mainly showed purulent changes (34.3%), tracheomalacia (27.1%), and positive pathogenic bronchoalveolar lavage fluid (25.4%). Older age, admission to the intensive care unit (ICU), positive modified Asthma Prediction Index (mAPI), and food allergy were risk factors for asthma. The odds ratio of mAPI was 4.066. The area under the receiver operating characteristic curve of the risk factors for predicting asthma was 0.8016.

**Conclusion:**

Wheezing phenotype and age may partly guide the etiological diagnosis of recurrent/persistent wheezing in infants and young children. Bronchoscopy is important for the diagnosis of refractory recurrent/persistent wheezing, while mAPI aids in the diagnosis of asthma. When infants and children with recurrent or persistent wheezing have the characteristics of older age, ICU admission, positive mAPI, and food allergy, the possibility of asthma may be considered.

## 1 Introduction

Wheezing is a common respiratory symptom in infants and young children, and one-third of children under the age of 3 years will have at least one episode of wheezing (1). In the 1994 Tuscon Prospective Study of Healthy Newborns, the incidence of wheezing in the first, second, and third years of life was found to be 32%, 17.3%, and 12.0%, respectively (2). In recent years, there has been an upward trend in the incidence of wheezing among infants in China (3).

The etiology of recurrent or persistent wheezing in infants and young children is complex, and the diagnosis is often difficult due to atypical disease characteristics, accompanying symptoms, and the limited number of available auxiliary tests (4). Thus far, no large-scale study has investigated the etiology and clinical features of recurrent or persistent wheezing in infants and young children. The only diagnostic guidelines available for recurrent or persistent wheezing in infants and young children were proposed by the American Thoracic Society in 2016 (5). No clear guidelines or expert consensus to guide the diagnosis and treatment of these children yet exists in China. In the absence of a clear etiological cause, clinicians use empirical treatment, which relieves the symptoms of the majority of affected children; however, approximately 20% of children still experience persistent or recurrent wheezing (6, 7).

Frequent hospitalization of infants and young children due to wheezing episodes seriously affects the children's health and lives, and places a considerable emotional and financial burden on their caregivers and families (8). Hence, the early identification of the underlying cause of recurrent or persistent wheezing followed by appropriate treatment can greatly improve the prognosis of the child and reduce the mental and financial burden on the patient's family. Therefore, we conducted a retrospective analysis of the etiology, clinical features, and auxiliary examinations among children under the age of 3 years with recurrent or persistent wheezing, and identified the risk factors for asthma to provide a clinical reference for diagnosis and management in this population.

## 2 Data and methods

### 2.1 Study population

We retrospectively enrolled a total of 612 infants and toddlers who were hospitalized for recurrent wheezing or persistent wheezing in Shenzhen Children's Hospital between 2019.09.01 and 2022.08.31. We included patients aged 0–36 months with acute episodes of wheezing diagnosed by a physician. Recurrent wheezing was defined as ≥3 cumulative wheezing episodes, with an asymptomatic interval of ≥1 week between episodes. Persistent wheezing was defined as wheezing symptoms lasting for ≥4 weeks. We excluded patients with breathing difficulties caused by upper airway disease.

### 2.2 Data collection

The following clinical data of the children were collected from the medical records system of our hospital: (a) basic information, such as gender, age, admission date, duration of hospitalization, and body mass index (BMI); (b) medical history, including family history of atopic, history of allergic diseases, history of underlying diseases, birth history, clinical manifestations, signs, and diagnosis; (c) results of laboratory tests, including routine blood tests, polymerase chain reaction (PCR) tests of nasopharyngeal swab for 13 pathogens of the respiratory tract (metapneumovirus, influenza A virus H3N2 and H1N1, influenza B virus, bocavirus, coronavirus, respiratory syncytial virus, adenovirus, rhinovirus, parainfluenza virus, *Mycoplasma*, and *Chlamydia*), sputum bacterial culture, and serum IgE tests for allergens [total IgE(tIgE); specific IgE(sIgE) antibodies for inhaled allergens, such as dust mites,pets furry,pollen, mould; sIgE tests for food allergens, such as milk, eggs, barley, nuts, shrimps and crabs]; (d) results of imaging studies, including chest x-ray, chest computed tomography (CT), and ultrasonography of the heart; (e) results of tidal lung-function tests, tidal fractional exhaled nitric oxide (FeNO) test, and bronchoscopy [microscopic changes, cytological results, and bronchoalveolar lavage fluid (BALF) pathogenetic results]; and (f) other relevant information.

### 2.3 Patient groups

The patients were grouped according to the wheezing types into the recurrent wheezing group (*n* = 486) and the persistent wheezing group (*n* = 126). Additionally, patients were divided into 3 groups according to their age at admission: (a) 0–12 months (*n* = 234), (b) 13–24 months (*n* = 236), and (c) 25–36 months (*n* = 142). Finally, patients were divided into 2 groups according to the etiology of the disease: the asthma group (*n* = 155) and the non-asthma group (*n* = 457).

The diagnosis of asthma was based on the Global Initiative for Asthma (GINA 2019) (9) criteria for diagnosing asthma in children under 5 years of age. Asthma was diagnosed if the child presented with all of the following characteristics: (a) there was a pattern of associated symptoms (i.e., wheezing, coughing, dyspnea/activity limitation) and recurrent episodes of nocturnal symptoms or awakenings; (b) there was a family history or personal history of atopic disease (such as history of food allergy, allergic rhinitis, or atopic dermatitis); and (c) asthma-controlling treatment was effective, and other diseases were excluded. In this study, the asthma group consisted of (a) children who had been clearly diagnosed with asthma in the past, and (b) children who were suspected to have asthma at the time of admission, and were diagnosed with asthma when inhaled glucocorticosteroid therapy was confirmed to be effective for at least 6 months after discharge from the hospital (confirmed via telephone follow-up). The remaining enrolled children formed the non-asthma group.

Recurrent wheezing associated with viral infections was diagnosed when the following conditions were met: (a) the duration of each wheezing episode was less than 10 days; (b) the intervals between wheezing episodes were completely normal (with no clinical sympyoms); (c) the conditions for suspected asthma were not present; and (d) other diseases were excluded. Children for whom this differential diagnosis could not be clarified during hospitalization were followed up for 6 months after discharge to clarify the final diagnosis.

The diagnosis of PBB was based on the criteria in the European Respiratory Society (ERS) statement on PBB in children (10). Specifically, PBB was diagnosed if the child presented with the following: (a) chronic wet cough (>4 weeks), (b) lower airway infection (recognized respiratory bacterial pathogens growing in sputum or BALF at a density of >10^4^ CFU ml^−1^ for a single bacterial species), and (c) cough resolved following a 2-week course of an appropriate oral antibiotic. Alternatively, PBB was clinically diagnosed based on the following criteria: (a) chronic wet cough (>4 weeks), (b) absence of symptoms or signs of other causes of wet or productive cough, and (c) cough resolved following a 2-week course of an appropriate oral antibiotic.

The diagnosis of tracheomalacia was based on bronchoscopy, either performed during this hospitalization or in the past.

The diagnosis of food allergy was made by combining the child's medical history, food-sIgE results, Skin prick tests, and the disappearance of symptoms after 2–4 weeks of avoidance of the suspected food while the reappearance of symptoms when it was reintroduced (11). Unfortunately, excitation experiment are not routinely conducted at our center. In our study, children with a definite diagnosis or a positive food-sIgE test were considered to have a food allergen sensitization. While children with a definite diagnosis or a positive inhalation-sIgE test were considered to have inhalation allergen sensitization.

### 2.4 Statistical analysis

Statistical analyses were carried out using SPSS v27.0.1 software. Categorical and continuous variables were presented as mean ± standard deviation, median, or proportions. The statistical significance of differences was assessed using the chi-square test (for categorical variables), and the Student test or the Mann–Whitney *U* rank-sum test (for continuous variables), depending on the distribution and variance of the data. Statistical significance was set at *P* < 0.05. Risk factors were considered for inclusion in the multivariate logistic regression analysis if they had a *P* value of <0.05 on univariate analysis.

### 2.5 Ethics statement

The study was approved by the ethics committee of Shenzhen Children's Hospital (Ethics No. 202311501). As this was a retrospective study, guardian consent was obtained on admission. During the follow-up telephone call for asthma diagnosis, verbal consent was obtained from the guardian. All study methods were performed in accordance with the ethical standards set out in the Declaration of Helsinki and its subsequent amendments.

## 3 Results

### 3.1 General characteristics

The male-to-female ratio in the total study population of 612 children was 3.3:1, with the proportion of boys being significantly higher than the proportion of girls (*P* < 0.001). The median age was significantly lower in the persistent wheezing group [10 (3, 33) months] than in the recurrent wheezing group [16 (3, 36) months; *P* < 0.05]. The majority of children in the persistent wheezing group were in the age group of 0–12 months (61.1%), while the majority of children in the recurrent wheezing group were in the age group of 13–24 months (42.4%). Both recurrent wheezing and persistent wheezing in infants and young children mainly occurred in the autumn and winter seasons (January, November, and December) in Shenzhen.

### 3.2 Etiology

The etiology of recurrent wheezing was as follows: recurrent wheezing associated with viral infections (48.4%), infantile asthma (28.0%), protracted bacterial bronchitis (PBB; 9.5%), tracheomalacia (4.9%), bronchopulmonary dysplasia (BPD; 4.1%), bronchiolitis obliterans (BO; 2.9%), tracheal stenosis (2.5%), pulmonary artery sling (0.8%), bronchial foreign body (0.6%), bronchiectasis (0.4%), postoperative esophageal atresia (0.4%), interstitial lung disease (ILD; 0.2%), gastroesophageal reflux (0.2%), primary immunodeficiency diseases (PIDs; 0.2%), neuromuscular diseases(0.2%), and inherited metabolic diseases (0.2%). The etiology of persistent wheezing was as follows: PBB (67.5%), tracheomalacia (22.2%), infantile asthma (15.9%), tracheal stenosis (4.8%), BO (2.4%), bronchial foreign body (1.6%), gastroesophageal reflux (1.6%), pertussis (1.6%), pulmonary artery sling (0.8%), BPD (0.8%), ILD (0.8%), and primary ciliary dyskinesia (PCD; 0.8%). The distribution of etiological diseases significantly differed between the recurrent wheezing and persistent wheezing groups (*P* < 0.05).

The distribution of the main etiological causes across different age groups（0–12 months, 13–24 months, 25–36 months）, was as follows: recurrent wheezing associated with viral infection (38.4%, 40.3%, 35.2%), infantile asthma (10.3%, 32.6%, 38.7%), PBB (32.9%, 14.8%, 13.4%), and tracheomalacia (15.4%, 5.9%, 1.4%). The specific subgroups are shown in Table 1.

**Table 1:** Comparison of the recurrent wheezing and persistent wheezing groups.

Etiology		Recurrent wheezing (*n*, %)				Persistent wheezing (*n*, %)				*P*
		Total (*n* = 486)	0–12 mo (*n* = 157)	13–24 mo (*n* = 206)	25–36 mo (*n* = 123)	Total (*n* = 126)	0–12 mo (*n* = 77)	13–24 mo (*n* = 30)	25–36 mo (*n* = 19)	
Infection-related	Recurrent wheezing associated with viral infections	235, 48.4	90, 57.3	95, 46.1	50, 40.7	–	–	–	–	<0.001
	PBB	46, 9.5	18, 11.5	18, 8.7	10, 8.1	85, 67.5	59, 76.6	17, 56.7	9, 47.4	<0.001
	Pertussis	2, 0.4	2, 1.3	–	–	2, 1.6	2, 2.6	–	–	0.189
Infantile asthma		136, 28.0	19, 12.1	71, 34.5	46, 37.4	20, 15.9	5, 6.5	6, 20.0	9, 47.4	0.005
Congenital abnormalities	Tracheomalacia	24, 4.9	14, 8.9	10, 4.9	–	28, 22.2	22, 28.6	4, 13.3	2, 10.5	<0.001
	Tracheal stenosis	12, 2.5	7, 4.5	4, 1.9	1, 0.8	6, 4.8	3, 3.9	2, 6.7	1, 5.3	0.288
	Pulmonary artery sling	4, 0.8	1, 0.6	3, 1.5	–	1, 0.8	1, 1.3	–	–	<1.000
–	Postoperative esophageal atresia	2, 0.4	–	1, 0.5	1, 0.8	–	–	–	–	<1.000
	BPD	20, 4.1	5, 3.2	5, 2.4	10, 8.1	1, 0.8	–	–	–	0.121
Other respiratory system diseases	BO	14, 2.9	5, 3.2	4, 1.9	5, 4.1	3, 2.4	1, 1.3	2, 6.7	–	<1.000
	ILD	1, 0.2	–	1, 0.5	–	1, 0.8	–	1, 3.3	–	0.370
–	Bronchiectasis	2, 0.4	–	2, 1.0	–	–	–	–	–	<1.000
	Bronchial foreign body	3, 0.6	1, 0.6	1, 0.5	1, 0.8	2, 1.6	1, 1.3	1, 3.3	–	0.601
Other systems	Gastroesophageal reflux	1, 0.2	1, 0.6	–	–	2, 1.6	–	2, 6.7	–	0.109
Systemic diseases	PCD	–	–	–	–	1, 0.8	1, 1.3	–	–	0.206
	PIDs	1, 0.2	–	1, 0.5	–	–	–	–	–	<1.000
	Inherited metabolic diseases	1, 0.2	–	1, 0.5	–	–	–	–	–	<1.000
	Neuromuscular diseases	2, 0.4	1, 0.6	–	1, 0.8	–	–	–	–	<1.000

PBB, protracted bacterial bronchitis; BPD, bronchopulmonary dysphasia; BO, bronchiolitis obliterans; ILD, interstitial lung disease; PCD, primary ciliary dyskinesia; PIDs, primary immunodeficiency diseases.

### 3.3 Clinical features

Statistically significant differences were found between the asthma and non-asthma groups in terms of clinical manifestations such as age, BMI, wheezing types, and the incidence of the three concave signs (*P* < 0.05). The remaining symptoms and signs did not significantly differ between the above 2 groups (*P* > 0.05; Table 2).

**Table 2:** Comparison of the asthma and non-asthma groups.

Items		Asthma group (*n* = 155)	Non-asthma group (*n* = 457)	*P*
General conditions	Age (months)	21.0 ± 7.6	15.7 ± 8.7	<0.001
	Hospitalization duration	4.2 ± 1.6	5.2 ± 3.0	<0.001
	BMI (kg/m^2^)	16.3 ± 2.3	17.0 ± 2.56	<0.001
Clinical features	Recurrent wheezing ratio (%)	87.1	76.8	0.006
	Family history of allergies (%)	32.9	23.0	0.019
	Food allergen sensitization (%)	50.3	28.4	<0.001
	Inhalant allergen sensitization (%)	25.8	8.1	<0.001
	Positive mAPI (%)	22.6	5.5	<0.001
	Fever (%)	42.6	46.8	0.401
	Wet cough (%)	87.1	90.4	0.287
	Triple concave sign (%)	32.9	17.9	<0.001
Infections	White blood cells (×10^9^/L)	11.7 ± 4.3	11.4 ± 4.3	0.357
	Neutrophil percentage (%)	53.2 (0.8, 92.2)	40.9 (1.3, 89.2)	<0.001
	CRP (mg/L)	5.5 (0, 138.3)	3.0 (0, 88.8)	<0.001
	Rhinovirus positive (%)	60.4	46.5	0.013
Allergies	Absolute peripheral blood eosinophil count (×10^9^/L) eosinophils (×109/L) peripheral blood (×109/L)	0.1 (0.0, 1.9)	0.1 (0.0, 1.6)	0.566
	Total serum IgE (IU/ml)	159.0 (2.5, 2,971.0)	58.3 (1.0, 2,421.0)	<0.001
	FeNO (ppb)	6 (3, 14)	13 (3, 24)	0.056
	BALF eosinophil proportion (%)	1.0 (0, 37.0)	0 (0, 27.0)	<0.001
Bronchoscopic manifestations	Purulent changes (%)	3.9	12.5	0.002
	Tracheomalacia (%)	1.3	10.5	<0.001
Treatment	Oxygen support (%)	36.8	25.2	0.007

BMI, body mass index; mAPI, modified asthma predictive index; CRP, C-reactive protein; IgE, immunoglobulin E; FeNO, fractional exhaled nitric oxide; ppb, parts per billion; BALF, bronchoalveolar lavage fluid.

Values are reported as percentages, mean ± standard deviation, and median (interquartile range).

### 3.4 Pathogenetics

PCR tests for 13 respiratory pathogens were performed in 588 (96.1%) children on admission. Positive results were obtained in 79.1% of the children tested. The most common pathogens identified were as follows: rhinovirus, 294 (50.0%); respiratory syncytial virus, 68 (11.6%); and parainfluenza virus, 53 (9.0%). Sputum bacterial cultures were carried out in 498 children, of whom, 258 (51.8%) children had positive culture results. The most commonly grown organisms included *Moraxella catarrhalis* (46.5%), *Streptococcus pneumoniae* (41.1%), and *Haemophilus influenzae* (26.0%).

A comparison of the mean leukocyte count, other indicators of infection, and the results of pathogen tests between the asthma and non-asthma groups is shown in Table 2. Significant differences were found in the neutrophil ratio, C-reactive protein level, and rhinovirus detection rates between the 2 groups (*P* < 0.05), whereas no statistically significant differences were observed in the leukocyte count, procalcitonin level, and detection rates of the rest of the pathogens tested between the 2 groups (*P* > 0.05).

### 3.5 Atopic characteristics

A total of 532 (86.9%) children had data on peripheral blood eosinophil counts. The absolute eosinophil count was greater than 0.15 × 10^9^/L [which indicates the presence of type 2 inflammation (9)] in 58 (37.4%) children in the asthma group and 188 (41.1%) children in the non-asthma group. A total of 161 (26.3%) children had data on BALF eosinophil proportion; the median value was 1.0% [0%, 37.0%] in the asthma group and 0% [0%, 27.0%] in the non-asthma group. Serum allergen testing was performed for 388 (63.4%) children. The results were as follows: elevated total immunoglobulin E (54.4%); positive for inhalant allergens, mainly dust mites (11.9%) and animal dander (6.4%); and positive food allergens, mainly cow's milk (24.0%), egg whites (15.2%), and wheat (3.6%). A total of 27 (4.4%) children underwent FeNO testing, and only 2 children in the non-asthma group were found to have a FeNO level higher than 20 ppb; the remaining 25 children had FeNO levels of less than 20 ppb.

The total IgE level, rate of food allergen sensitization and inhalant allergen sensitization, and BALF eosinophil proportion significantly differed between the asthma and non-asthma groups (*P* < 0.05), while no statistically significant differences were found in the absolute eosinophil count in the peripheral blood and FeNO results between the 2 groups (*P* > 0.05; Table 2). The prevalence of allergic rhinitis, allergic conjunctivitis, and atopic dermatitis did not significantly differ between the asthma and non-asthma groups (*P* > 0.05).

### 3.6 Lung imaging and function

Chest imaging was performed for a total of 565 (92.3%) children, and the predominant imaging manifestations were exudative changes (59.3%), lung-marking thickening (35.2%), and other manifestations, including BO, BPD, unequal inflation, tracheal bronchus, airway stenosis, interstitial changes, pulmonary atelectasis, and pleural effusion. Pulmonary arterial slings were found in 4 children on contrast-enhanced chest CT and cardiac ultrasonography. No statistically significant differences in imaging manifestations were found between the asthma and non-asthma groups (*P* > 0.05).

Tidal breathing flow volume curve testing was completed in 38 children (16 in the asthma group, 22 in the non-asthma group), and the bronchodilation test was completed in 5 children. Among them, 35 (92.1%) children were found to have obstructive ventilatory dysfunction (6 mild, 19 moderate, and 10 severe), and 1 child had a positive diastolic test. No significant difference in the rate of obstructive conditions was detected between the asthma and non-asthma groups (*P* > 0.05).

### 3.7 Bronchoscopy

Bronchoscopy was performed for 181 (29.6%) children, and showed the following results: purulent changes (34.3%), tracheomalacia (27.1%), tracheal stenosis (6.1%), distal occlusion (4.4%), foreign body (1.7%), plastic bronchitis (0.6%), positive pathogenic BALF (25.4%), and positive oil red O staining of BALF (3.3%). Bronchoscopy was performed in 32 (20.6%) children in the asthma group and 149 (32.6%) children in the non-asthma group. The proportion of microscopic purulent changes and tracheomalacia significantly differed between the 2 groups (*P* < 0.05), whereas the number of nucleated cells, macrophages, and pathogen-detection rate did not differ between the 2 groups (*P* > 0.05). Tracheoscopy was performed in 86 out of 131 children with PBB, and they all had bacterial cultures, of which, 20 patients (23.3%) had a positive result.

### 3.8 Risk factors for asthma

**Figure 1:**
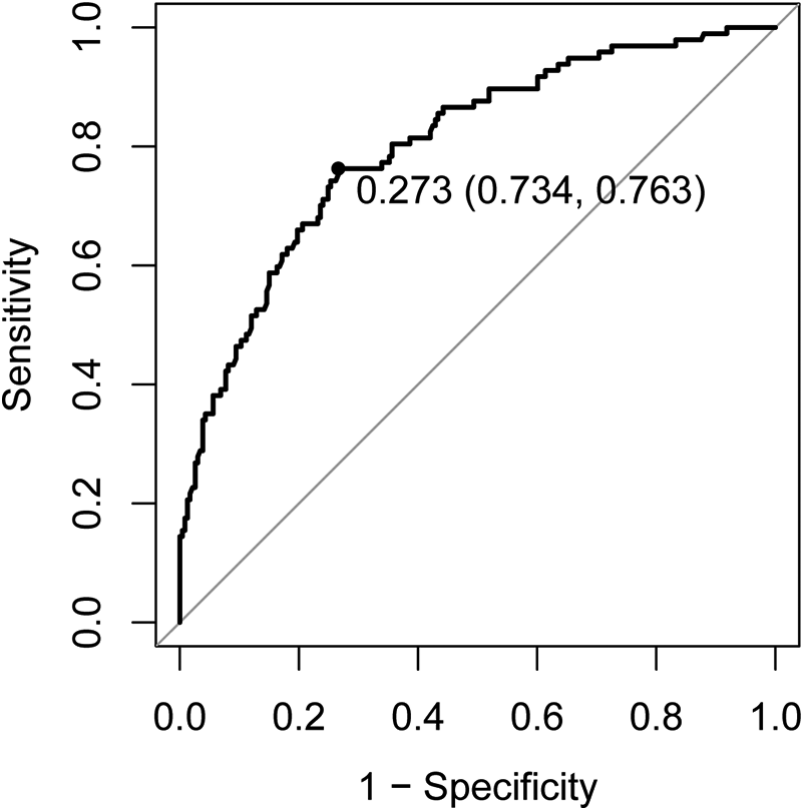
Receiver operating characteristic curve of risk factors (older age, ICU admission, positive mAPI, and food allergen sensitization) and protective factor (hospitalization duration) for asthma. ICU, intensive care unit; mAPI, modified asthma prediction index.

Logistic regression analysis of the aforementioned differential factors led to the conclusion that age, intensive care unit (ICU) admission, positive modified Asthma Predictive Index (mAPI), and food allergen sensitization were risk factors for asthma, while hospitalization duration was a protective factor for asthma (Table 3). The area under the ROC curve (AUC) of the above five factors for predicting asthma was 0.8016 (Figure 1).

**Table 3:** Multivariate regression analysis of risk factors for asthma.

Factors	B	Wald	OR (95% CI)	*P*
Intercept	−2.343	16.64	0.096 (0.03–0.29)	<0.001
Age	0.074	18.841	1.077 (1.042–1.115)	<0.001
Hospitalization duration	−0.189	5.372	0.827 (0.7–0.965)	0.020
ICU admission	2.452	4.391	11.613 (1.576–240.978)	0.036
Positive mAPI	1.403	9.935	4.066 (1.735–10.066)	0.002
Food allergen sensitization	0.704	5.782	2.021 (1.141–3.607)	0.016
Rhinovirus detection	0.453	2.363	1.573 (0.885–2.819)	0.124
Oxygen support	0.59	3.277	1.805 (0.948–3.421)	0.070

OR, odds ratio; CI, confidence interval; ICU, intensive care unit; mAPI, modified asthma predictive index.

## 4 Discussion

In this study, the demographic characteristics of children with different wheezing types and different etiologies were analyzed, and it was found that boys accounted for 77.0% of patients with recurrent or persistent wheezing, which was significantly higher than the proportion of girls, suggesting that the prevalence of recurrent or persistent wheezing among infants and young children is highest among boys. This result is consistent with the finding that boys are more prone to wheezing in studies such as the Tucson study (2). We found a significant age difference in children with different wheezing types, with the mean age of children with persistent wheezing being significantly less than that of children with recurrent wheezing. This result was considered to be related to differences in the main etiological factors of the different wheezing types. The age distribution of the different causes of wheezing showed that 2 causes, namely, PBB and developmental anomalies, were predominant in the persistent wheezing group, and both these conditions were most common in children aged 0–12 months. In the recurrent wheezing group, the number of children with infantile asthma gradually increased from the age of 13 months onwards. Therefore, age may guide the consideration of different wheezing types and etiologies.

Among the etiologies of recurrent or persistent wheezing in infants and children, the main clinical causes that have been clearly associated with wheezing in previous studies include infantile asthma, recurrent respiratory infections, gastroesophageal reflux, foreign body aspiration, PBB, tracheomalacia, tracheal stenosis, BO, tuberculosis, congenital heart disease, cystic fibrosis, PCD, vascular slings, BPD, and immunodeficiency diseases (12, 13). In this study, in order of prevalence, these etiologies were as follows: (a) infections (including recurrent wheezing associated with viral infections, PBB, and pertussis); (b) asthma in infants and young children; (c) developmental anomalies (including tracheomalacia, tracheal stenosis, BPD, and pulmonary artery sling); (d) BO; (e) bronchial foreign body; (f) digestive disorders (including gastroesophageal reflux and postoperative esophageal atresia); and (g) other diseases such as PCD, ILD, and PIDs, which is consistent with the above studies.

The causes of recurrent wheezing and persistent wheezing differed somewhat, with the top 3 causes of recurrent wheezing being recurrent wheezing associated with viral infections, infantile asthma, and PBB, while the top 3 causes of persistent wheezing being PBB, tracheomalacia, and infantile asthma. The proportion of asthma in the persistent wheezing group was not low. In clinical practice, clinicians treating children with persistent wheezing need to be alert to the possibility of asthma. In the recurrent wheezing group, the proportion of children with wheezing caused by infectious factors (including recurrent wheezing associated with viral infections and PBB) decreased with age. In the persistent wheezing group, PBB and tracheomalacia accounted for a higher percentage of children in the age group of 0–12 months. The proportion of children with infantile asthma significantly increased with age in both the recurrent and persistent wheezing groups. These results suggest that in infants and toddlers, the phenotype of wheezing and the age at presentation can provide some guidance for etiological diagnosis and initial treatment.

In a previous study of bronchoscopy in children with refractory recurrent or persistent wheezing, inflammatory changes in the lower respiratory tract were found in 49.5% of patients, and anatomical abnormalities were found in 45.7% of patients, with tracheomalacia being the most common anatomical alteration (14). Similarly, another study of children with wheezing found bacterial lower respiratory tract infections in 48.3% of patients and tracheomalacia in 20.4% of patients on bronchoscopy (15). In the present study, microscopic septic changes and anatomical abnormalities were found in 34.1% and 37.6% of patients, respectively, with tracheomalacia being the most common anatomical change. These results are consistent with those of the previous studies, and indicate a high detection rate of bronchoscopy in children with recurrent or persistent wheezing, confirming the important role of bronchoscopy in the etiological diagnosis of these conditions.

The diagnosis of asthma in infants and young children is often challenging because recurrent or persistent wheezing is also common in infants and young children without asthma, and it is not possible to routinely assess their airflow limitation or bronchodilator responsiveness (16). In the present study, the risk factors for asthma were analyzed to predict the diagnosis of asthma. The mean age of the patients was greater and the BMI was lower in the asthma group than in the non-asthma group. The proportions of patients with recurrent wheezing and the triple concave sign were significantly higher in the asthma group. A history of allergy, a family history of allergy, total IgE level, BALF eosinophil proportion, and the positive mAPI rate were higher in the asthma group, which is consistent with the findings of previous studies (17).

Among the pathogenic findings, the 3 most common viruses detected were rhinovirus, respiratory syncytial virus, and parainfluenza virus, in that order. The 3 most common bacteria were *Moraxella catarrhalis*, *Streptococcus pneumoniae*, and *Haemophilus influenzae*, in that order. *Moraxella catarrhalis* mostly colonized the respiratory tract, and the detection rates of the other bacteria were essentially the same as those in previous findings of respiratory pathogen surveillance in China (18). Among all the pathogens tested, only the rhinovirus detection rate was significantly higher in the asthma group than in the non-asthma group; no differences were found in the detection rates of the rest of the pathogens. This finding was attributed to the lower proportion of viral infections in the non-asthma group than in the asthma group, and the much higher incidence of rhinovirus infection than that of the other pathogens. No differences in lung function and FeNO values were found between the asthma and non-asthma groups, which is inconsistent with previous studies (19). This difference may be related to the small sample size of patients who underwent these 2 tests. Bronchoscopic manifestations such as purulent changes and tracheomalacia were significantly more common in the non-asthma group than in the asthma group, which was attributed to the predominant etiological factors of infections and airway developmental abnormalities in the non-asthma group.

In 2018, the Chinese Society of Childhood Asthma proposed the following diagnostic model for asthma in children up to the age of 6 years: wheezing episodes with a cumulative frequency of ≥4 (3 points), presence of reversible airflow limitation (3 points), presence of allergic rhinitis and/or atopic dermatitis (1 point), history of allergy in first-degree relatives (1 point), positive test results for internal or external allergens (1 point), dermatitis (1 point), and a positive allergen test result (1 point). A total score of ≥4 is diagnostic for asthma. However, only one multicenter study in China has validated this model as being stable, sensitive, and specific; the application of this model requires further validation and optimization (20, 21). In our study, the above diagnostic conditions did differ between the asthma and non-asthma groups. However, multivariate regression analysis of the characteristics that were significantly different between the 2 groups showed that age, ICU admission, positive mAPI, and food allergen sensitization were independent risk factors for asthma, which is slightly different from the above model. The risk of asthma increased by 0.08 times for every year of age. The risk of asthma was 10.61 times higher for children admitted to the ICU than for children not requiring ICU admission. The risk of asthma was 1.02 times higher among children with food allergen sensitization than among children without.it. For each additional day of hospitalization, the risk of asthma decreased by 0.17 times. The diagnosis of asthma can be considered in the presence of these characteristics in children with recurrent or persistent wheezing, but further validation is also required.

Currently, mAPI is often used as an important factor for predicting the development of asthma in infants and children. Studies have shown that infants and children with a positive mAPI are 2.6–5.5 times more likely to develop asthma than infants and children who have a negative mAPI; however, the sensitivity and positive predictive value of mAPI are limited (22, 23). The regression analysis in the present study showed that mAPI had an OR of 4.066, which is in line with the results of the previous studies, and to a certain extent, affirms its diagnostic predictive value for asthma.

### 4.1 Limitations

In this study, only inpatients were included, which may introduce selection bias. The follow-up time in this study was short, and it will not be possible to observe the regression of wheezing, allergies, lung-function test results, and FeNO changes in most of the included children until they have reached school age.

### 4.2 Summary

This study analyzed the distribution of the etiological causes of recurrent and persistent wheezing, and explored the risk factors associated with the diagnosis of asthma, which can provide some guidance for clinical work. Nonetheless, many shortcomings remain. Thus, further research is needed.

## Data Availability

The original contributions presented in the study are included in the article/Supplementary Material, further inquiries can be directed to the corresponding authors.
